# Angiogenesis Suppression via VEGF–VEGFR2 Inhibition and Stromal–Endothelial Crosstalk Disruption by Myrosinase-Activated Broccoli Extract

**DOI:** 10.3390/molecules31061042

**Published:** 2026-03-20

**Authors:** Irina Naletova, Alfonsina La Mantia, Giuseppe Antonio Malfa, Simone Bianchi, Donata Arena, Valeria Di Domenico, Francesco Attanasio, Claudia Di Giacomo, Barbara Tomasello

**Affiliations:** 1 Institute of Crystallography, CNR, Via P. Gaifami 18, 95126 Catania, Italy; irina.naletova@ic.cnr.it; 2 Department Drug and Health Sciences, University of Catania, Viale A. Doria 6, 95125 Catania, Italy; alfy.lamantia@gmail.com (A.L.M.); gmalfa@unict.it (G.A.M.); simone.bianchi@unict.it (S.B.); valeriadidomenico20@gmail.com (V.D.D.); cdigiaco@unict.it (C.D.G.); 3 Research Centre on Nutraceuticals and Health Products (CERNUT), University of Catania, Viale A. Doria 6, 95125 Catania, Italy; 4 Department of Agriculture, Food and Environment, University of Catania, Via Valdisavoia 5, 95123 Catania, Italy; donata.arena@unict.it

**Keywords:** HUVEC, angiogenin, fibroblasts, migration, vessel formation, *Brassica oleracea* var. *italica*, collagen, ROS

## Abstract

Dysregulated angiogenesis is involved in cancer and numerous ischemic, autoimmune and inflammatory diseases, prompting extensive research that has yielded a growing array of angiogenesis-modulating molecules used in clinical practice. The dietary phytocomplex of Cruciferous vegetables exhibits multiple biological activities in both in vitro and in vivo models. However, the impact of a myrosinase-activated broccoli extract (MaBE) on angiogenesis, as well as on stromal–endothelial interactions governing endothelial cell behavior, has not yet been explored. We investigated the effects of MaBE on endothelial–stromal crosstalk using endothelial cells (HUVECs) and fibroblasts (HFF1) both individually and in a fibroblast-conditioned medium model. MaBE dose-dependently inhibited endothelial viability, migration and tube formation, key steps of angiogenesis, through interference with the VEGF–VEGFR2 axis. Notably, MaBE also markedly suppressed HFF1-driven HUVEC migration and capillary-like structure formation, likely through the inhibition of fibroblast motility and the downregulation of VEGF and angiogenin signaling in HFF1 cells. Overall, these findings provide new insight into MaBE regulation of pro-angiogenic behaviors in both endothelial cells and fibroblasts while disrupting their functional interplay. By targeting multiple cellular compartments and key mediators involved in angiogenesis, MaBE emerges as a promising bioactive extract with potential relevance for the management of pathological angiogenesis-related disorders.

## 1. Introduction

Angiogenesis is a profoundly anabolic physiological process by which new blood capillaries form from pre-existing ones and occurs throughout life in both health and disease [1].

Angiogenic processes involve a complex and dynamic interaction between endothelial cells (ECs) and the tissue microenvironment and need to be finely balanced during development and adulthood. Aberrant angiogenesis, excessive or insufficient, contributes, directly or indirectly, to different pathologies, ranging from cancer, autoimmune diseases, macular degeneration, and retinopathy to impaired repair of ischemic tissues, coronary heart disease and wound healing [2,3,4].

Under physiological conditions, the balance of positive and negative angiogenic modulators within the vascular microenvironment is fundamental for the development and growth of new capillaries in angiogenic processes [5] and requires the functional activities of a number of molecules, including angiogenic factors, extra-cellular matrix proteins, adhesion receptors, and proteolytic enzymes [6,7,8].

The most commonly described angiogenic growth factors and cytokines include vascular endothelial growth factor (VEGF) [9], fibroblast growth factor (FGF), tumor necrosis factor-alpha (TNF-α), transforming growth factor-beta (TGF-β) [6], and angiogenin [10].

Vascular endothelial growth factor (VEGF) is widely recognized as the principal driver of angiogenesis. VEGF regulates a coordinated sequence of events, including endothelial cell proliferation, basement membrane remodeling, migration, and motility, which collectively underlie new vessel formation [11].

The VEGF family comprises several members, among which VEGF-A plays a dominant role in physiological and pathological angiogenesis by activating VEGFR-1 and VEGFR-2 on endothelial cells [12]. Although VEGFR-1 displays higher ligand affinity, VEGFR-2 is the primary signaling receptor mediating VEGF-induced angiogenic responses [13]. Ligand binding triggers receptor dimerization and phosphorylation, activating downstream pathways that promote endothelial proliferation, migration, and vascular permeability [14].

While VEGF is essential for vascular homeostasis, its dysregulation drives pathological angiogenesis and vascular permeability in multiple diseases [15], including cancer progression and metastasis [16] and retinal disorders such as age-related macular degeneration and diabetic or hypertensive retinopathy [17,18].

In addition to VEGF, angiogenin (ANG) is a potent regulator of angiogenesis, capable of directly inducing neovascularization and acting as a permissive factor for VEGF-driven angiogenic responses [19,20]. ANG is a secreted growth factor broadly expressed in human tissues and fluids, where it regulates key cellular processes including proliferation, migration, and invasion [21,22]. Dysregulated ANG expression has been associated with tumor growth and progression [23,24] and with wound-associated neovascularization and tissue repair [25]. Given its broad expression and functional pleiotropy, ANG contributes to both physiological and pathological angiogenesis, reinforcing the concept that vascular homeostasis depends on a tightly regulated balance between pro- and anti-angiogenic factors.

Beyond soluble angiogenic factors such as VEGF and angiogenin, angiogenesis is critically regulated by the tissue microenvironment, which consists of a dynamic interplay between cellular and non-cellular components responsible for maintaining organ homeostasis [26,27]. Under pathological conditions, reciprocal crosstalk between cells and the surrounding microenvironment drives extracellular matrix remodeling, fibroblast activation, and the recruitment of immune and inflammatory cells, ultimately stimulating endothelial cells and promoting angiogenesis through the coordinated release of cytokines, chemokines, and growth factors [28,29].

In this context, fibroblasts have emerged as key regulators of both physiological and pathological angiogenesis. By actively remodeling the extracellular matrix and locally supplying pro-angiogenic mediators, fibroblasts support endothelial cell survival, migration, and organization into functional capillary networks, thereby acting as central orchestrators of angiogenesis at the microenvironmental level [30].

Collectively, these observations underline angiogenesis as a microenvironment-driven process and indicate that effective control of pathological neovascularization may require interventions targeting not only endothelial cells but also stromal components and their reciprocal interactions. Current anti-angiogenic therapies predominantly aim to interfere with individual signaling nodes within the angiogenic cascade using synthetic or recombinant agents [31,32]; however, such approaches may be limited by the emergence of resistance, toxicity or reduced efficacy over time [33,34]. These considerations support the exploration of multi-compartment strategies, including bioactive phytocomplexes, also capable of modulating endothelial–stromal crosstalk.

A wide range of plant-derived compounds has attracted increasing attention due to their beneficial effects on human health [35,36,37]. Among these, glucosinolates (GLs) and their hydrolysis products, isothiocyanates (ITCs), exert pleiotropic biological activities through multiple molecular mechanisms, ultimately displaying anti-inflammatory, antioxidant, and chemoprotective properties [38]. While research has historically focused on their anticancer effects, accumulating evidence indicates that cruciferous vegetables also influence cardiometabolic, neurological, and musculoskeletal disorders [39,40]. In particular, sulforaphane, one of the best-characterized ITCs, exhibits anti-angiogenic activity by interfering with key steps of neovascularization, including pro-angiogenic signaling, extracellular matrix remodeling, and endothelial proliferation, migration, and tube formation [41,42].

Despite these advances, the effects of myrosinase-activated phytocomplex from *Brassica oleracea* var. *italica* (Broccoli), in short myrosinase-activated broccoli extract (MaBE), on angiogenesis, especially with respect to signaling interactions between endothelial cells and fibroblasts, remain poorly understood. In the present study, we evaluated the impact of MaBE on both endothelial and fibroblast functions and their cross-talk by setting up a 3D collagen gel model in which primary human umbilical vein endothelial cells (HUVECs) were exposed to conditioned medium from MaBE-treated fibroblasts. This approach allowed us to dissect how MaBE modulates stromal–endothelial communication during angiogenesis. Our findings demonstrate that MaBE exerts significant anti-angiogenic activity and, for the first time, reveal its ability to disrupt endothelial–fibroblast interactions, highlighting its potential relevance for the development of novel anti-angiogenic strategies.

## 2. Results and Discussion

### 2.1. Extract Characterization and Working Solution

The methanolic extraction from leaves of *Brassica oleracea* var. *italica* (BE) yielded 4.8% starting from 100 g of fresh plant material, with a total glucosinolate content (TGC) of 125.52 ± 0.50 µmol glucoraphanin equivalents per gram of extract (µmol GE/g), as determined by the spectrophotometric method (Table 1). This amount corresponds to approximately 6.02 ± 0.36 µmol GE per gram of fresh leaves, calculated based on the extraction yield.

**Figure 1:**
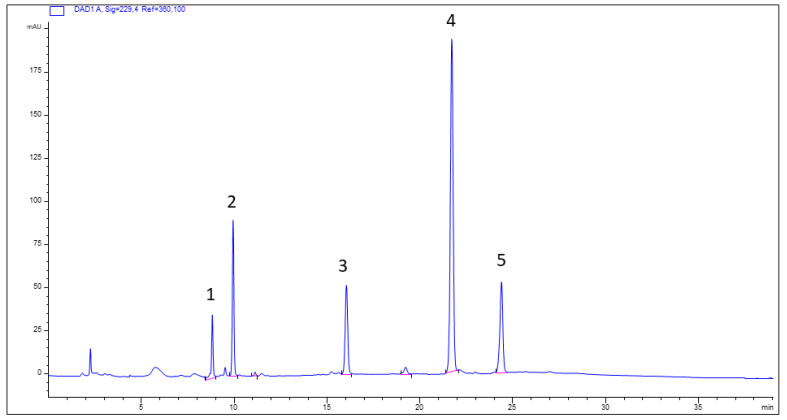
HPLC-DAD phytochemical fingerprint of BE. Column: Kinetex C18, 25 cm × 4.6 mm, 5 µm d.m. The numbers indicating peaks refer to the identified compounds reported in Table 2.

The qualitative phytochemical profile of BE was further examined using HPLC-DAD analysis. Five compounds were identified from the trace recorded at 230 nm (Figure 1 and Figure S1), comprising two principal constituents (peaks 2 and 4) and three minor peaks. Peak 1 corresponded to sinigrin, peak 2 to glucoraphanin, peak 3 to gluconapin, peak 4 to glucobrassicin, and peak 5 to neoglucobrassicin (Figure 1, Figures S2 and S3 and Table 2).

For experimental procedures, aqueous working solutions of the extract (20 mg/mL) with myrosinase (T4528-25UN, Sigma-Aldrich, Milan, Italy) (0.5 mg/mL, corresponding to 300 mU) were freshly prepared (MaBE) and diluted in culture medium to obtain the desired final concentrations, expressed as % *v*/*v*.

Recovering broccoli (*Brassica oleracea* var. *italica*) by-products, such as leaves, offers a sustainable way to convert agro-industrial waste into glucosinolate-rich nutraceutical ingredients [43]. After harvest, broccoli leaves remaining on plants retain high levels of glucosinolates, which remain a valuable resource for supporting the circular economy. When glucosinolates are converted by myrosinase into bioactive forms, they can provide several health benefits [43]. In broccoli by-products, glucoraphanin and glucobrassicin are the primary representatives among glucosinolates (up to 60% of the total glucosinolates) [44]. The cold methanol-based extraction protocol used in this study effectively preserved glucosinolate stability from hydrolysis to isothiocyanates during extraction, demonstrating comparable recovery rates to those obtained with hot methanol-based extraction [45,46], as shown by the yield % of crude extract and the total glucosinolate content, quantified spectrophotometrically (Table 1). For cell culture assays, sterile aqueous working solutions freshly prepared with the extract and exogenous myrosinase at 300 mU/mL enable controlled hydrolysis of bioactive compounds and ensure uniform treatment of cell cultures.

### 2.2. Cytotoxicity Assessment of MaBE Extract on HUVECs

**Figure 2:**
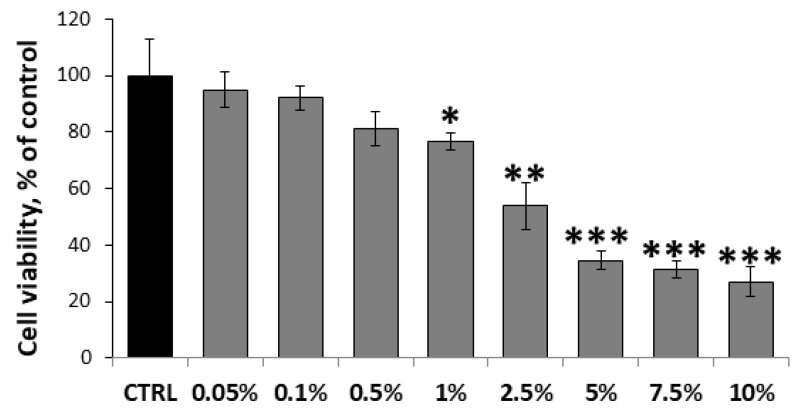
Dose–response experiment of viability (MTT assay) on HUVECs. Cells were incubated for 24 h with MaBE at different concentrations: 0.05–10% in full DMEM medium supplemented with 1% of FBS. Results are presented as mean ± SD (*n* = 4) and normalized with respect to the untreated control cells. One-way ANOVA with Tukey’s test was used for statistical analysis; * *p* ≤ 0.05, ** *p* ≤ 0.01, *** *p* ≤ 0.001 vs. untreated control cells.

To select appropriate concentrations for subsequent functional studies, a preliminary viability experiment was conducted on HUVECs using MaBE across a concentration range of 0.05% to 10% *v*/*v* (1.25–2.51 nmol GE/mL). Cell viability was evaluated via the MTT assay after 24 h of incubation. The extract proved safe up to 0.5%, with no significant effects on viability. Moderate cytotoxicity emerged only at 1%, resulting in a 24 ± 3% reduction in viability, indicative of the onset of cytotoxic effects. Cytotoxicity increased in a dose-dependent manner with higher concentrations, reaching approximately 66–74% viability loss at 5–10% (Figure 2).

The observed dose-dependent cytotoxicity suggests that bioactive components in the MaBE extract, including glucosinolates or sulforaphane derivatives, exert direct or indirect damage to essential cellular pathways in endothelial cells. The 1% threshold marks a critical point where viability loss becomes statistically significant, escalating rapidly at higher doses, which aligns with prior reports on glucosinolates derivatives-induced endothelial stress via various action mechanisms [40,47].

Remarkably, this dose–response profile confirms the broccoli extract’s biocompatibility at low concentrations, making it suitable for endothelial cell studies. For follow-up functional assays evaluating the extract’s antiangiogenic potential (e.g., tube formation, migration), cytocompatible doses (0.1–1% *v*/*v* corresponding to 2.51–25.1 nmol GE/mL) were selected to ensure cellular functionality while probing therapeutic efficacy.

### 2.3. Anti-Angiogenic and Antimigration Capacities of MaBE Extract on HUVECs

**Figure 3:**
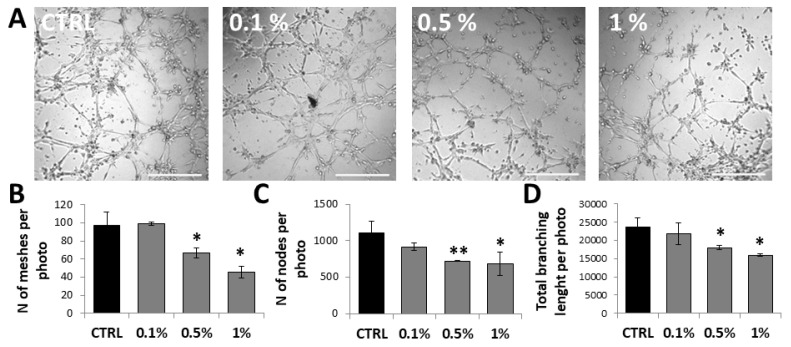
Inhibitory effect of MaBE on HUVEC capillary-like network formation. Representative images of tube formation (**A**) and quantification of meshes/photo (**B**), nodes/photo (**C**) and Total branching length/photo (**D**) after 3 h in HUVECs treated with broccoli extract (0.1, 0.5 and 1%). Images of tube formation were acquired using an inverted microscope (scale bar 1000 µm and magnification 4×), and the number of meshes was quantified with ImageJ software. Values represent mean ± SD (*n* = 3). One-way ANOVA with Tukey’s test was used for statistical analysis; * *p* ≤ 0.05, ** *p* ≤ 0.01 vs. untreated control.

To assess the MaBE anti-angiogenic potential, a tube formation assay was performed on HUVECs exposed to 0.1%, 0.5%, and 1% concentrations during network formation for 3 h. In the control wells, seeded HUVECs rapidly adhered to the matrigel and migrated across the gel, extending their morphology and developing lateral protrusions that enabled cell–cell interactions within 3 h. Conversely, the extract exhibited dose-dependent inhibition of capillary network formation, with maximal effects at 1% (45 ± 6 meshes/field) and markedly reduced versus untreated controls (97 ± 15 meshes/field) (Figure 3A,B). The same trend was observed for the number of nodes (684 ± 159) and the total branching length (15,940 ± 377) (Figure 3C,D).

**Figure 4:**
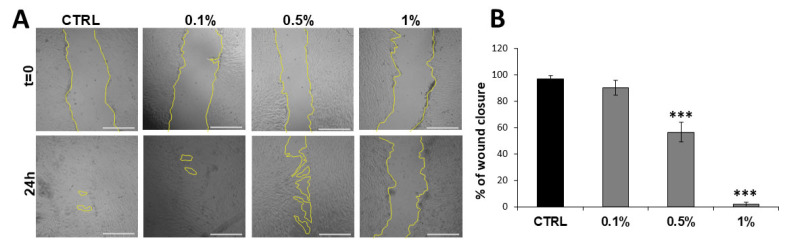
Inhibitory effect of MaBE on HUVEC migration. (**A**) Representative images of HUVECs cultured to confluence in DMEM, mechanically wounded (t = 0), and subsequently exposed to MaBE in DMEM containing 1% FBS for 24 h (scale bar 1000 μm; magnification 4×). The yellow lines indicate the borders of the cellular monolayers. (**B**) Quantitative analysis of changes in the wound (cell-free) area relative to baseline (t = 0). Data are expressed as mean ± SD (*n* = 3) and normalized to untreated control cells. Statistical analysis was performed using one-way ANOVA followed by Tukey’s post hoc test; *** *p* ≤ 0.001 versus untreated control cells.

Given that tube formation is closely linked to endothelial cell migration, a wound healing assay was conducted, involving standardized wound creation in HUVEC monolayers and quantification of closure rates via directed cell movement. At 0.5%, migration was markedly slowed with a wound closure rate of 56 ± 7%; at 1%, migration was completely inhibited, preventing monolayer recovery (1 ± 1%) (Figure 4A,B).

Angiogenesis is a multistep process involving the release of angiogenic factors, their binding to receptors on endothelial cells (ECs), endothelial activation, migration and proliferation, extracellular matrix (ECM) remodelling, and ultimately tube formation [3].

Only a limited number of studies have investigated the effects of glucosinolates and their metabolites on angiogenesis. Jackson et al. demonstrated that sulforaphane suppresses angiogenesis in endothelial cell cultures by disrupting microtubule polymerization and inhibiting mitotic progression and also reduces neovascularization in vivo in a VEGF-impregnated Matrigel mouse model [41]. In cancer models, particularly liver and prostate cancer, sulforaphane has been shown to target multiple metabolic pathways involved in tumour progression, including the inhibition of angiogenesis, suppression of proliferation, and induction of apoptosis [40,48]. Similarly, other isothiocyanates (ITCs), such as benzyl isothiocyanate (BITC), have been reported to inhibit invasion and angiogenesis in human glioma cells [49], while phenethyl isothiocyanate (PEITC) interferes with angiogenesis through modulation of HIF family proteins [50].

In line with these reports, the present study demonstrates that MaBE significantly attenuates the angiogenic phenotype of human endothelial cells. MaBE-treated HUVECs exhibit both impaired migration and reduced formation of capillary-like structures compared with untreated controls. The dose-dependent reduction in mesh number suggests that MaBE may interfere with VEGF signaling and/or ECM remodeling processes essential for tube morphogenesis. Moreover, the complete blockade of endothelial migration at 1% MaBE is consistent with previous evidence showing that sulforaphane-rich extracts disrupt actin cytoskeleton dynamics and matrix metalloproteinase (MMP) expression in various cell types.

Since endothelial sprouting is a driving force of pathological neovascularization, the observed anti-angiogenic and anti-migration effects of MaBE highlight its potential even as an anti-tumor agent. By limiting new vessel formation, MaBE may effectively restrict tumor nutrient supply and reduce metastatic dissemination, reinforcing angiogenesis inhibition as a cornerstone of modern anticancer strategies.

### 2.4. MaBE Extract Interferes with the VEGF–VEGFR2 Axis and VEGF Release

**Figure 5:**
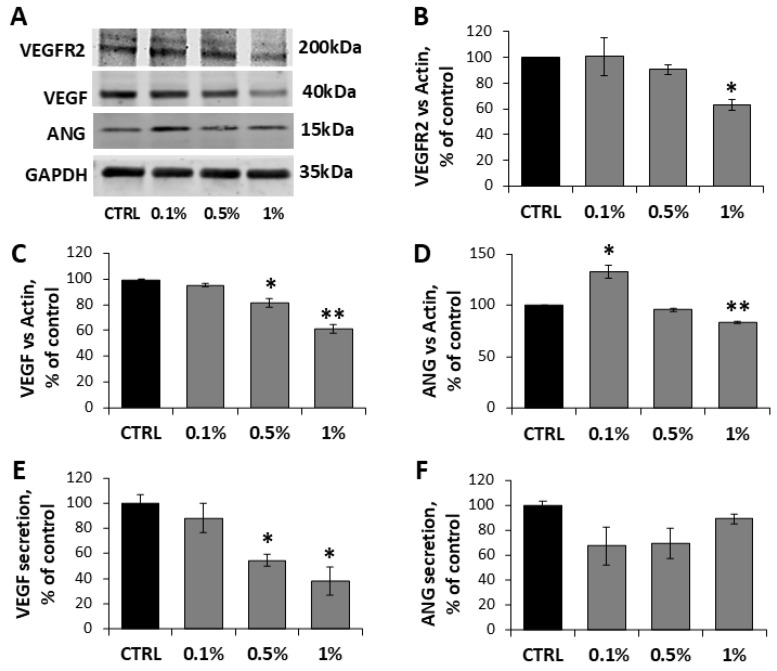
MaBE disrupts angiogenesis signaling in HUVECs. (**A**) Representative Western blot images and densitometric analysis of VEGFR2 (**B**), VEGF (**C**) and ANG (**D**) in HUVECs incubated with MaBE extract (0.1, 0.5 and 1%) for 24 h. The expression level of proteins is normalized to GAPDH and reported as a percentage of control cells. (**E**,**F**) VEGF and ANG release in culture medium is expressed as % of control. Values represent mean ± SD (*n* = 3). One-way ANOVA with Tukey’s test was used for statistical analysis; * *p* ≤ 0.05, ** *p* ≤ 0.01, vs. untreated control.

To elucidate the molecular basis of the MaBE anti-angiogenic effects, we examined its impact on the VEGF-VEGFR2 axis and the expression/release of pro-angiogenic factors (VEGF and Ang) in HUVECs. At 1% concentration, VEGFR2 expression was reduced to 63 ± 4% (Figure 5A,B). This was accompanied by dose-dependent downregulation of VEGF expression (95 ± 2%, 81 ± 3%, 61 ± 3% reduction at 0.1%, 0.5%, 1%, respectively) (Figure 5A,C) and marked inhibition of extracellular VEGF release (81 ± 3%) (Figure 5E). By contrast, angiogenin (ANG) showed a less pronounced decrease, with only modest reductions in its expression at 1%, while its secretion was unchanged (Figure 5A,D).

Angiogenesis is driven by coordinated signaling from multiple growth factors, among which VEGF–VEGFR2 represents the central axis regulating endothelial cell survival, migration, permeability, and extracellular matrix remodeling [37]. Dysregulated VEGF signaling drives diverse pathological conditions, including tumor angiogenesis, cardiovascular diseases, and ocular disorders [11]. Disruption of this pathway effectively impairs endothelial activation and vascular sprouting. In this context, the hydrolyzed broccoli extract primarily targets VEGF signaling, as indicated by the concurrent reduction in VEGF availability and VEGFR2 expression, thereby weakening both autocrine and paracrine endothelial stimulation.

Although angiogenin also contributes to angiogenesis via VEGF-dependent manner, in endothelial cells, this factor exerts its pro-angiogenic function primarily after nuclear translocation, where it stimulates rRNA transcription and supports cell proliferation [20]. The modest modulation of angiogenin observed at the expression level, and not significantly altered in its extracellular release, suggests that the extract does not broadly suppress angiogenic mediators but instead selectively interferes with VEGF-driven mechanisms. In addition, in line with the literature, we may speculate that our extract could interfere with angiogenin nuclear trafficking rather than its synthesis or secretion, thereby limiting its pro-angiogenic activity in HUVECs and contributing to the MaBE effect; however, this aspect needs to be further explored.

Consistent with previous evidence on plant-derived polyphenols and sulforaphane-containing extracts, which have been shown to disrupt angiogenesis through modulation of transcriptional programs, epigenetic regulation, and intracellular signaling pathways [37,41,42,47,51], the observed effects align with a targeted anti-angiogenic mechanism responsible for the suppression of endothelial proliferation, signaling and neovessel formation, thus explaining the anti-angiogenic properties of the hydrolyzed broccoli extract observed in this study.

Overall, selective disruption of the VEGF–VEGFR2 axis appears to be the dominant mechanism underlying the anti-angiogenic activity of the extract, positioning it as a potential complementary strategy to VEGF-centred anti-angiogenic approaches.

### 2.5. MaBE Extract Selectively Impairs Fibroblast Migration Without Early Cytotoxicity

Beyond ECs, the tissue microenvironment also includes fibroblasts, which play a pivotal role in stromal remodelling and angiogenesis. To investigate the effects of MaBE on fibroblasts, HFF-1 cells were exposed for 24 h to increasing concentrations of the extract (up to 10%), and cell viability (MTT assay), morphology, intracellular redox status (ROS production), migratory ability (wound healing assay) and collagen type I alpha 1 (COL1A1) expression were assessed.

**Figure 6:**
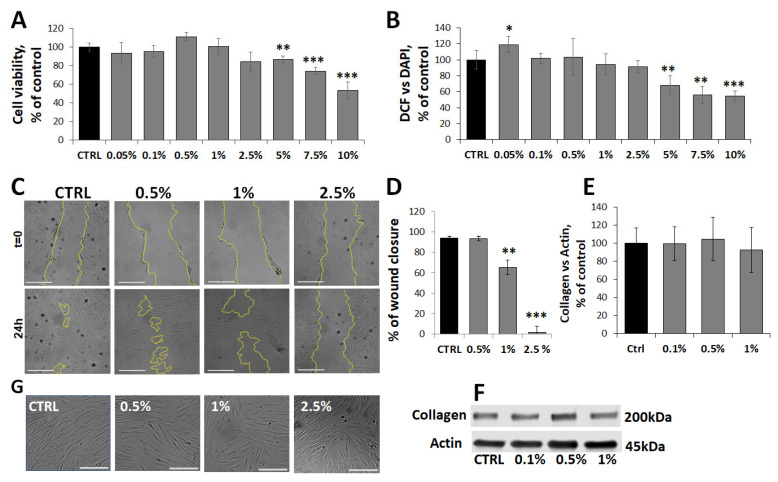
Effects of MaBE on HFF-1 Fibroblast Viability, Redox Status, and Migration. (**A**,**B**) Dose–response analysis of cell viability (MTT assay (**A**)) and intracellular ROS production (DCF assay, (**B**)) in HFF-1 cells following 24 h exposure to MaBE (0.05–10%). (**C**) Representative phase-contrast images of confluent HFF-1 monolayers subjected to scratch wounding (t = 0) and treated with MaBE for 24 h. Scale bar: 500 μm; magnification: 4×. The yellow lines indicate the borders of the cellular monolayers. (**D**) Quantitative evaluation of wound closure expressed as changes in the cell-free area relative to baseline (t = 0). (**E**) Densitometric analysis and (**F**) representative Western blot image of Collagen1A1 in HFF-1 cells incubated with MaBE extract (0.1, 0.5 and 1%) for 24 h. (**G**) Representative images of HFF1 cell morphology acquired by optical inverted light microscopy. Original magnification 10×, scale bar 200 μm. The expression level of protein is normalized to actin and reported as a percentage of control cells. Data are shown as mean ± SD (*n* = 3) and normalized to untreated control cells. Statistical significance was determined by One-way ANOVA followed by Tukey’s post hoc test; * *p* ≤ 0.05, ** *p* ≤ 0.01 and *** *p* ≤ 0.001 versus untreated controls.

Unlike HUVECs, HFF-1 fibroblasts exhibited a marked resistance to MaBE treatment. No significant changes in cell viability or intracellular ROS levels were observed at concentrations up to 2.5% (Figure 6A,B). At higher concentrations (5–10%), a dose-dependent cytotoxic effect became evident (14 ± 3%, 26 ± 4%, 46 ± 9% reduction at 5%, 7.5%, and 10%, respectively), likely associated with mitochondrial dysfunction, as indicated by a concomitant reduction in both cell viability and ROS production, which reached approximately 50% of control values at the highest concentration tested.

Notably, fibroblast migratory capacity, a key functional hallmark, was strongly affected at lower, non-cytotoxic concentrations. Treatment with 1% MaBE reduced wound closure by 35 ± 7% while exposure to 2.5% completely abolished cell migration, resulting in persistent open wound areas (2 ± 1%) (Figure 6C,D). Conversely, COL1A1 production was not significantly affected by MaBE treatment at these concentrations (Figure 6E,F), suggesting that the extract does not markedly interfere with extracellular matrix production under the experimental conditions employed. These functional effects were accompanied by subtle morphological alterations at 2.5%, including reduced cell elongation, irregular contours, and increased intercellular spacing, consistent with impaired migratory behaviour (Figure 6G).

Fibroblasts’ activation and migration, both involved in tissue remodeling and angiogenesis within both physiological and pathological microenvironments, are controlled by growth factor signaling (e.g., PDGF, FGF, EGF) and redox/inflammatory pathways. In this framework, natural products have emerged as modulators of fibroblast behavior, capable of either promoting tissue repair or preventing excessive stromal activation [52].

The present findings indicate that MaBE exerts a selective functional effect on fibroblasts by markedly suppressing migratory capacity through mechanisms independent of ROS modulation, possibly interfering with cytoskeletal organization or signaling pathways governing cell movement. Given the pivotal role of fibroblast migration in stromal expansion and angiogenic support, this functional inhibition may have relevant implications in pathological contexts.

The ability of phytochemicals to regulate fibroblast migration has been widely documented, although their effects are highly context-dependent. Curcumin, resveratrol, and berberine have been associated with enhanced fibroblast function during normal wound repair, whereas bufalin, lycorine, homoharringtonine, and hyperforin display anti-migratory and anti-fibrotic activities in pathological settings [53,54]. These dual actions highlight the capacity of phytochemicals to fine-tune fibroblast responses rather than inducing nonspecific toxicity. Glucosinolate-derived metabolites, particularly sulforaphane, have been mainly studied for their anti-migratory effects in cancer cells, where they modulate epithelial–mesenchymal transition, cytoskeletal organization and adhesion dynamics [55]. Our previous work further demonstrated that sulforaphane-based formulations impair migration and adhesion in astrocytoma cells by disrupting cytoskeletal architecture and integrin α5-dependent signaling [56]. More recently, sulforaphane has been shown to reprogram fibroblast secretory activity, preventing pathological cardiac remodeling [57]. Together, these observations support the notion that our glucosinolate-containing phytocomplex can exert fine regulatory effects on fibroblast motility function without inducing evident toxicity, potentially involving focal adhesion turnover, actin dynamics, or matrix metalloproteinase regulation.

Activated fibroblasts are known to secrete a wide range of ECM components and matricellular proteins, including collagens, fibronectin, SPARC, tenascin, and connective tissue growth factor, which collectively support endothelial morphogenesis and angiogenesis [58]. Although previous work identified fibroblast-derived collagen I as a contributor to endothelial lumen formation [30], the absence of MaBE-induced modulation of COL1A1 expression suggests that the Brassica-derived phytocomplex does not substantially interfere with collagen production at the transcriptional level. Nevertheless, this represents a limitation of the study, as it does not exclude effects on other ECM components, collagen secretion, post-translational modifications, or matrix organization. Moreover, the experimental time frame and concentration range selected may not have been optimal to capture more subtle or delayed changes in ECM remodeling.

Overall, the fibroblast-specific effects observed here complement the endothelial-targeted anti-angiogenic activity of MaBE, including inhibition of VEGF/VEGFR2 signaling and tube formation. The combination of strong anti-migratory activity with minimal cytotoxicity highlights a cell-type-selective mode of action, supporting the potential of MaBE as a multi-compartment modulator of stromal dynamics in pathological angiogenesis.

### 2.6. MaBE Modulates Angiogenic Cross-Talk Between Endothelial Cells and Fibroblasts

To assess the role of fibroblasts in angiogenesis and its modulation by MaBE, endothelial cells were treated with conditioned medium from fibroblasts (FCM) exposed to MaBE at concentrations non-cytotoxic for HUVECs.

**Figure 7:**
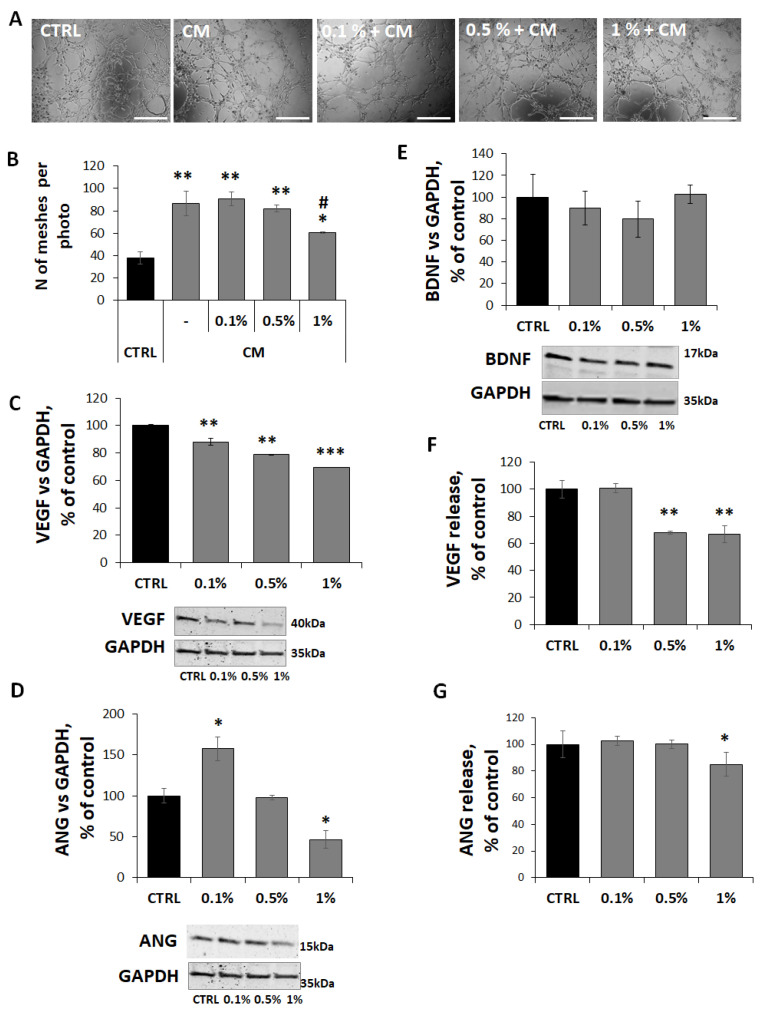
MaBE impairs fibroblast-driven angiogenesis. (**A**) Representative images of endothelial tube formation and (**B**) quantification of the number of meshes after 3 h in HUVECs treated with FCM exposed to MaBE extract (0.1, 0.5, and 1%), diluted 1:1 with M200 medium. Tube formation images were acquired using an inverted microscope (magnification 4×; scale bar: 1000 µm), and the number of meshes was quantified using ImageJ software. Representative Western blot images and corresponding densitometric analyses of VEGF (**C**), ANG (**D**), and BDNF (**E**) protein expression in HFF1 cells incubated with MaBE extract (0.1, 0.5, and 1%) for 24 h. Protein expression levels were normalized to GAPDH and expressed as a percentage of untreated control cells. (**F**,**G**) VEGF and ANG release in FCM is expressed as % of control. Data are presented as mean ± SD (*n* = 3). Statistical analysis was performed using One-way ANOVA followed by Tukey’s post hoc test; * *p* ≤ 0.05, ** *p* ≤ 0.01, *** *p* ≤ 0.001 vs. untreated control; # *p* ≤ 0.01 vs. cells treated with CM.

Results showed that FCM markedly promoted angiogenic responses in endothelial cells, enhancing both endothelial cell sprouting and lumen formation, as indicated by a significant increase in branch formation (101 ± 13) and mesh number (87 ± 11) compared with HUVECs cultured in standard M200-supplemented medium (69 ± 3 and 38 ± 6, respectively) (Figure 7).

Notably, MaBE treatment, particularly at 1%, counteracted the pro-angiogenic paracrine signaling of FCM, significantly impairing tubulogenesis and vascular network organization and reducing mesh formation by 39 ± 1. These results indicate that MaBE extract disrupts fibroblast-mediated angiogenic support (Figure 7A,B).

To elucidate the molecular mediators underlying this effect, the expression and release of key pro-angiogenic factors were examined, together with the neurotrophin BDNF. Consistently, analysis of pro-angiogenic mediators revealed a dose-dependent downregulation of VEGF expression, with a 31% ± 1 reduction at 1% MaBE (Figure 7C) as well as a lower VEGF release (67% ± 6) compared with FCM alone (Figure 7G). Moreover, angiogenin expression was markedly decreased (54% ± 11) at the same concentration, with a concomitant reduction in its release (85% ± 9) (Figure 7D,F), whereas BDNF levels remained unaffected (Figure 7E).

Intercellular communication within the tissue microenvironment plays a pivotal role in angiogenesis and is mediated through both direct cell–cell interactions and paracrine signaling among stromal and endothelial components [59,60]. Despite increasing recognition of the importance of stromal cells in vascular remodeling, the molecular crosstalk between fibroblasts and endothelial cells remains incompletely understood.

In the present study, we investigated the impact of HFF1 fibroblasts on the angiogenic behavior of HUVECs and evaluated the ability of MaBE extract to modulate this interaction. Our findings demonstrate that FCM strongly enhances endothelial tubulogenesis, as reflected by increased branching and mesh formation, resembling cancer-associated fibroblasts (CAFs) behavior [61]. This pro-angiogenic effect was significantly attenuated when fibroblasts were pre-treated with MaBE, indicating that the extract disrupts fibroblast-mediated angiogenic support.

In addition to modulating angiogenic signaling together with cell migration, MaBE appeared to interfere with direct fibroblast–endothelial interactions, impairing endothelial recruitment and vascular network organization. These observations suggest that MaBE affects both soluble factor release and contact-dependent mechanisms involved in fibroblast–endothelial communication. A previous study reported that EC tube formation in three-dimensional (3D) fibrin gels is much more strictly dependent on the distance between the EC and the fibroblasts than on the distance between the EC and the media [62].

The pronounced angiogenic activity induced by fibroblast-conditioned medium further supports the central role of fibroblasts in actively shaping a pro-angiogenic microenvironment through the production of angiogenic mediators and ECM components [30]. In several pathological conditions, fibroblasts have been reported to overexpress multiple pro-angiogenic factors, thereby contributing to aberrant or dysfunctional angiogenesis at disease sites [50,60,63]. Among these factors, angiogenin and, most notably, VEGF-A have been identified as key paracrine mediators, making angiogenic signaling pathways attractive therapeutic targets [11,64]. Indeed, inhibition of angiogenesis has proven effective across various disease contexts, with cancer being the most extensively investigated [33,64]. In this regard, MaBE treatment effectively reprogrammed the fibroblast pro-angiogenic phenotype, inducing a marked and dose-dependent reduction in VEGF and angiogenin levels while leaving BDNF expression unaffected. This selective modulation indicates that MaBE preferentially interferes with VEGF-dependent angiogenic pathways rather than broadly suppressing fibroblast-derived trophic support. Consistently, several natural compounds have also been identified as CAF-targeting agents capable of modulating specific signaling pathways and exerting anti-angiogenic effects alongside anticancer activity. Notable examples include conophylline (*Ervatamia microphylla*), fraxinellone (*Dictamnus dasycarpus*), and mangostin (*Garcinia mangostana*), which have demonstrated efficacy in limiting angiogenesis in digestive system tumor progression [65,66,67].

To our knowledge, evidence of fibroblast-mediated anti-angiogenic effects exerted by *Brassicaceae olereacea*-derived phytocomplex hydrolyzed by myrosinase is currently lacking. However, comparable anti-angiogenic activity has been reported in other experimental settings for specific isothiocyanates, such as sulforaphane. In particular, Liu et al. (2017) demonstrated that sulforaphane inhibited HepG2-induced migration, adhesion, and tube formation of HUVECs by suppressing STAT3, HIF-1α, and VEGF expression [55]. In line with these observations, our data extend current knowledge by highlighting the ability of MaBE to modulate angiogenesis indirectly through the reprogramming of fibroblast–endothelial communication.

## 3. Materials and Methods

### 3.1. Plant Material and Extraction Procedure

The plant material was provided by the Horticultural and Floricultural Crop Biotechnology research group of the Department of Agriculture, Food and Environment, University of Catania. The cultivation method is detailed in Arena et al. [42]. Leaves of a commercial variety of broccoli (*Brassica oleracea* L. var. *italica*, Brassicaceae), Cavolo Broccolo Ramoso Calabrese (S.A.I.S. S.p.A. seed company, Cesena, Italy), were collected immediately after the harvest of inflorescences at the end of November, after an average of 135 days from sowing. To ensure representative sampling, leaves at multiple vegetative growth stages were collected from several individual plants. A voucher specimen (11/23) of the plant material was deposited at the Department of Drug and Health Sciences, University of Catania. After collection, leaves were gently washed, surface-dried, and snap-frozen at −80 °C. 100 g of frozen leaves were ground in liquid nitrogen and extracted by maceration (1:10 *w*/*v*) with 80% (*v*/*v*) methanol at 4 °C for 60 min, with continuous stirring, three times [68]. The combined extracts were filtered, concentrated tenfold under reduced pressure using a rotary evaporator, and lyophilized to obtain a dry residue.

### 3.2. Total Glucosinolate Content

The total glucosinolate content in the *Brassica oleracea* var. *italica* extract (BE) was quantified according to the method described by Gallaher et al. [69]. The assay relies on the alkaline hydrolysis of glucosinolates, leading to the formation of 1-thioglucose, which reduces ferricyanide [Fe(CN)_6_]^3−^ to ferrocyanide [Fe(CN)_6_]^4−^, resulting in a decrease in yellow coloration. Briefly, the extract was dissolved in 1 M NaOH and incubated at room temperature for 30 min. The obtained solution was subsequently neutralized with 7.2% *v*/*v* concentrated HCl (37% *v*/*v*) and combined with a 2 mM K_3_[Fe(CN)_6_] solution prepared in 0.4 M phosphate buffer at pH 7.0 in a 1:1 ratio. After two minutes, absorbance was recorded at 420 nm. Glucoraphanin was used as the calibration standard, and the results were expressed as µmol glucoraphanin equivalents per gram of extract (µmol GE/g extract).

### 3.3. Determination of Glucosinolates by HPLC-DAD

The GLS extraction was carried out according to the ISO 9167-1:1992 protocol [70], with slight modifications. A total of 5 mg of the sample was dissolved in 5 mL of 70% methanol and then filtered through 0.22 μm syringe filters before analysis. GLS desulfation was conducted using a gravity-flow column packed with glass wool and 0.5 mL of DEAE Sephadex A-25 resin (GE17-0170-01, Merk Life Science S.r.l, Milan, Italy), pre-equilibrated with 0.02 M acetate-pyridine buffer. A 2 mL volume of extract was added to the column, followed by 0.5 mL of 0.02 M pyridine solution to maintain a basic pH. For enzymatic desulfation, 15 µL of sulfatase (E.C. 3.1.6.1, Type H1 from Helix pomatia, Sigma-Aldrich, St. Louis, MO, USA; CAS No. S9626-5KU) was added. The columns were left at room temperature overnight. Desulfo-GLSs were then eluted with 1.5 mL of ultrapure water and analyzed by HPLC using an Agilent 1200 Series system with a diode-array detector (DAD) set at 229 nm and a Kinetex C18 column (250 × 4.6 mm, 5 µm particle size). The mobile phase used solvent A (ultrapure water) and solvent B (acetonitrile:water, 20:80, *v*/*v*). The gradient program was as follows: 1% B at 1 min, increased to 99% B by 21 min, held at 99% B until 24 min, returned to 1% B at 29 min, and maintained until 39 min, with a total runtime of 39 min. The flow rate was set at 0.8 mL min^−1^, and the injection volume was 20 µL.

Individual glucosinolates were identified by matching their retention times and UV spectra to external standards from ChromaDex (Santa Ana, CA, USA). The standards included sinigrin, glucoraphanin, gluconapin, glucobrassicin, neoglucobrassicin.

### 3.4. Cell Culture Maintenance and Treatments

Human umbilical vein endothelial cells (HUVECs, CRL-1730 ™) and human foreskin fibroblasts (HFF-1, SCRC-1041) were obtained from ATCC (LGC Standards S.r.L., Milan, Italy). HUVECs were maintained in Vascular Cell Basal Medium (ATCC-LGC Standards S.r.L., Milan, Italy) supplemented with the Endothelial Cell Growth Kit-BBE (ATCC-LGC Standards S.r.L., Milan, Italy) and streptomycin (100 μg/mL, Sigma-Aldrich-Saint Louis, MO, USA) and were used at passages ≤ 5. HFF-1 cells were cultured in high-glucose DMEM containing 4 mM L-glutamine, 1 mM sodium pyruvate, 15% fetal bovine serum (FBS, Sigma-Aldrich, Saint Louis, MO, USA), and streptomycin (100 μg/mL).

Both cell lines were cultured in tissue culture–treated Corning^®^ flasks (Sigma-Aldrich, St. Louis, MO, USA) at 37 °C in a humidified incubator with 5% CO_2_. For experimental procedures, cells were seeded 24 h before the assays at densities optimized for each experimental condition. Aqueous stock solutions of MaBE (20 mg/mL, myrosinase 300 mU) were freshly prepared and diluted in culture medium to obtain the desired final concentrations (% *v*/*v*), and cells were treated for 24 h.

### 3.5. Cytotoxicity Assays

Cell viability was assessed at 70–80% confluence following 24 h exposure to MaBE at concentrations ranging from 0.05% to 10%. Viable cells were quantified using the MTT assay, based on the reduction of MTT (3-(4,5-Dimethylthiazol-2-yl)-2,5-diphenyltetrazolium bromide) (Sigma-Aldrich, St. Louis, MO, USA) to formazan. After 90 min of incubation, the reaction was terminated by the addition of DMSO (Sigma-Aldrich, St. Louis, MO, USA), and absorbance was measured at 569 nm using a VICTOR Nivo Multimode plate reader (PerkinElmer, Shelton, CT, USA). Results are expressed as the percentage of viable cells relative to untreated controls for each concentration [71,72].

### 3.6. ROS Production

HFF-1 cells were exposed to MaBE at concentrations ranging from 0.05% to 10% for 24 h. Intracellular reactive oxygen species (ROS) levels were assessed by staining cells with 2′,7′-dichlorofluorescein (DCF). Fluorescence was measured using a VICTOR Nivo Multimode spectrophotometer (PerkinElmer, Shelton, CT, USA) at excitation/emission wavelengths of 493/523 nm. Hoechst 33,342 staining (excitation/emission: 361/497 nm) was used to normalize DCF fluorescence to cell number in each well. Data are expressed as the relative increase in DCF fluorescence compared with untreated control cells [73].

### 3.7. In Vitro Angiogenesis Assay on Matrigel^®^ for Tube Formation

Matrigel^®^ Basement Membrane Matrix (Corning^®^, Sigma-Aldrich, St. Louis, MO, USA) was thawed at 4 °C on ice for 18 h prior to use. A thin coating of Matrigel^®^ (100 μL per well) was applied to 48-well plates and allowed to polymerize at 37 °C for 30 min. HUVECs (3 × 10^4^ cells/well) were seeded onto the solidified matrix, and MaBE was gently added immediately after cell plating. Tube formation was monitored, and images were captured after 3 h using phase-contrast microscopy. Quantitative analysis of tubular structures was performed by determining the number of meshes per field using ImageJ software 1.53 g (https://imagej.net/ij/ (accessed on 16 March 2026).; Java 1.8.0_112 (64 bit)) [74].

### 3.8. Scratch Wound Closure Assay

Confluent monolayers of HFF1 cells or HUVECs grown in 24-well Corning^®^ tissue culture plates (Sigma-Aldrich, St. Louis, MO, USA) were mechanically wounded at time zero (t_0_) using a sterile 200-μL pipette tip and subsequently washed with PBS to remove detached cells. Reference marks were placed on the plates immediately after scratching to ensure that the same wound area was imaged throughout the experiment. Cells were then treated with MaBE for 24 h in complete medium supplemented with 1% FBS at concentrations ranging from 0.5% to 2.5% depending on the cell line selected. Serial phase-contrast images were acquired using an inverted microscope, Evos FL (Life Technologies, Grand Island, NY, USA) equipped with a 4× objective. Quantitative analysis of cell migration was performed using ImageJ software by calculating the percentage of wound closure relative to T_0_ [74].

### 3.9. Collection of Fibroblast-Conditioned Medium for Angiogenesis Assays

Subconfluent HFF1 cells cultured in 24-well Corning^®^ tissue culture plates (Sigma-Aldrich, St. Louis, MO, USA) were exposed for 24 h to DMEM supplemented with 1% FBS and MaBE at concentrations of 0.1%, 0.5%, and 1%. After treatment, the conditioned medium (FCM) was collected, clarified by centrifugation, and diluted 1:1 with basal HUVEC medium immediately prior to use in angiogenesis experiments.

### 3.10. Direct and Sandwich ELISA Assay

Culture media were collected after 24 h of treatment with MaBE, centrifuged at 14,000× *g* for 10 min, and the resulting supernatants were transferred to clean microtubes and stored at −80 °C until analysis.

The concentration of vascular endothelial growth factor (VEGF) released into the culture medium was quantified using a sandwich ELISA. Briefly, polyvinyl chloride (PVC) microtiter plates (Santa Cruz Biotechnology, Inc, Dallas, TX, USA) were coated overnight at 4 °C with 5 µg/mL of capture antibody (anti-VEGF, code PAB12284) diluted in carbonate–bicarbonate buffer (pH 9.6). Plates were then washed with PBS and blocked with 1% BSA in PBS for 2 h at room temperature. After washing, samples were added and incubated for 6 h at 4 °C. Plates were subsequently washed and incubated overnight at 4 °C with 4 µg/mL of detection antibody (anti-VEGF, code sc-7269). Following additional washes, plates were incubated for 1 h with an HRP-conjugated secondary antibody (sc-516102) [75].

Angiogenin (ANG) levels in culture supernatants were determined using a direct ELISA. PVC microtiter plates (Santa Cruz Biotechnology, Inc -Dallas, TX, USA) were coated overnight at 4 °C with culture medium samples. Plates were washed, blocked with 1% BSA in PBS for 2 h at room temperature, and then incubated overnight at 4 °C with 5 µg/mL of detection antibody (anti-ANG, code sc-74528). After washing, plates were incubated for 2 h with an HRP-conjugated secondary antibody (sc-516102).

For both assays, immunoreactivity was visualized using TMB substrate solution (Sigma-Aldrich, St. Louis, MO, USA). The reaction was allowed to develop for 15 min and was subsequently stopped with TMB stopping solution (Sigma-Aldrich, St. Louis, MO, USA). Absorbance was measured at 450 nm using a VICTOR Nivo Multimode plate reader (PerkinElmer, Shelton, CT, USA).

### 3.11. Protein Extraction and Western Blotting Analysis

For protein expression analysis, HUVECs and HFF1 cells were seeded in tissue culture–treated multiwell plates (Sigma-Aldrich, St. Louis, MO, USA) and allowed to reach approximately 90% confluence before treatment with MaBE at concentrations ranging from 0.1% to 1%. Following treatment, cells were lysed using RIPA buffer (Sigma-Aldrich, St. Louis, MO, USA) supplemented with protease and phosphatase inhibitor cocktails. Cells were immediately scraped, transferred to microcentrifuge tubes, and incubated on ice for 30 min.

Lysates were clarified by centrifugation at 13,000 rpm for 10 min, and the resulting supernatants were collected. Total protein concentration was determined using the Bradford assay, with bovine serum albumin (BSA) used to generate the standard curve. Equal amounts of protein were separated by SDS–PAGE using 4–20% precast gels (Mini-PROTEAN, Bio-Rad, Hercules, CA, USA) and subsequently transferred onto nitrocellulose membranes. To reduce nonspecific binding, membranes were blocked using a commercial blocking buffer (LI-COR Biosciences, Lincoln, NE, USA) and then incubated overnight at 4 °C with the appropriate primary antibodies (Table 3).

Membranes were incubated at room temperature for 45 min with IRDye^®^ 680 or IRDye^®^ 800–conjugated goat anti-rabbit or anti-mouse secondary antibodies (1:20,000; LI-COR Biosciences, Lincoln, NE, USA). Immunoreactive bands were visualized using the Odyssey CLx Infrared Imaging System (LI-COR Biosciences, Lincoln, NE, USA). Quantitative densitometric analysis was performed with ImageJ software (version 1.53 g; National Institutes of Health, USA). Band intensities were expressed as arbitrary densitometric units (A.D.U.), normalized to GAPDH levels, and reported as percentages relative to untreated control cells [76].

### 3.12. Data and Statistical Analysis

Results are reported as mean ± standard deviation from at least three independent experiments, each performed with three biological replicates. Statistical comparisons among multiple groups were conducted using one-way ANOVA followed by Tukey’s multiple-comparison post hoc test. All analyses were carried out using GraphPad Prism version 9.0 (GraphPad Software Inc., La Jolla, CA, USA). A *p* value < 0.05 was considered statistically significant.

## 4. Conclusions

This study identifies MaBE as an effective modulator of pathological angiogenesis through a dual, cell-type–selective mechanism involving both endothelial and stromal compartments. In endothelial cells, MaBE strongly inhibits angiogenic sprouting by selectively disrupting the VEGF–VEGFR2 signaling axis, leading to reduced VEGF availability, VEGFR2 downregulation, impaired migration, and collapse of capillary-like structures. This targeted interference suggests a multi-hit anti-angiogenic activity that may complement VEGF-centered approaches while potentially limiting resistance.

Importantly, MaBE also modulates stromal contributions to angiogenesis by selectively reducing fibroblast motility. We also report for the first time that MaBE trans-inhibits endothelial angiogenic activity by disrupting fibroblast–endothelial crosstalk, a key driver of aberrant neovascularization.

Together, these findings highlight MaBE as a multi-compartment regulator of abnormal angiogenesis, extending its potential relevance beyond cancer to other angiogenesis-driven disorders like fibrosis, diabetic retinopathy, and rheumatoid arthritis. The combination of endothelial targeting and stromal modulation supports further translational investigation of MaBE as a bioactive extract with potential applicability in angiogenesis-related pathologies.

## Figures and Tables

**Table 1:** Yield and total glucosinolate content (TGC) of *Brassica oleracea* var. *italica* extract.

	Yield (%)	TGC (µmol GE/g)
*B.oleracea* var. *italica*	4.8	125.52 ± 0.50

**Table 2:** Compounds identified in BE by HPLC-DAD.

Peak	Compound	Wavelength (nm)	Ret. Time (min.)
1	Sinigrin	230	8.81
2	Glucoraphanin	230	9.93
3	Gluconapin	230	16.05
4	Glucobrassicin	230	21.73
5	Neoglucobrassicin	230	24.40

**Table 3:** Antibodies used for protein detection.

Antibody	Code	Company	Dilution
VEGF	ab229377	Abcam	1:1000
ANG	sc-74528	SantaCruz	1:500
VEGFR2	sc-6251	SantaCruz	1:500
COL 1A1	#91144	Cell Signaling	1:1000
BDNF	ab108319	Abcam	1:2000
Actin	#4970	Cell Signaling	1:2000

Abcam Inc, Waltham, MA, USA; Santa Cruz Biotechnology, Inc, Dallas, TX, USA; Cell Signaling Technology, Inc., Danvers, MA, USA.

## Data Availability

The original contributions presented in this study are included in the article/Supplementary Material. Further inquiries can be directed to the corresponding authors.

## Supplementary Materials

The following supporting information can be downloaded at: https://www.mdpi.com/article/10.3390/molecules31061042/s1, Figure S1: HPLC-DAD chromatograms of desulfo-GLSs standards (A) and desulfo-GLSs *Brassica oleracea* var. *italica* extract (B); Figure S2: Chemical structures of identified compounds in BE; Figure S3: UV spectra of the standard compound alone or identified in BE.
