# Supplementary material for: Angiogenesis Suppression via VEGF–VEGFR2 Inhibition and Stromal–Endothelial Crosstalk Disruption by Myrosinase-Activated Broccoli Extract
Source: Molecules. 2026 Mar 20;31(6):1042. doi: 10.3390/molecules31061042 (PMC13028631; doi:10.3390/molecules31061042)
Supplement: Supplementary file 1 [file molecules-31-01042-s001.zip › molecules-4170115-supplementary.pdf]

Supplementary Materials

Article

Angiogenesis Suppression via VEGF–VEGFR2 Inhibition and Stromal–Endothelial Crosstalk Disruption by Myrosinase-Activated Broccoli Extract

Irina Naletova [1], Alfonsina La Mantia [2], Giuseppe Antonio Malfa [2,3], Simone Bianchi [2,3], Donata Arena [4], Valeria Di Domenico [2], Francesco Attanasio [1,*], Claudia Di Giacomo [2,3,†] and Barbara Tomasello [1,2,3,*,†]

[1] Institute of Crystallography, CNR, via P. Gaifami 18, 95126 Catania, Italy; irina.naletova@ic.cnr.it
[2] Department Drug and Health Sciences, University of Catania, Viale A. Doria 6, 95125 Catania, Italy; alfy.lamantia@gmail.com (A.L.M.); gmalfa@unict.it (G.A.M.); simone.bianchi@unict.it (S.B.); valeriadidomenico20@gmail.com (V.D.D.); cdigiaco@unict.it (C.D.G.)
[3] Research Centre on Nutraceuticals and Health Products (CERNUT), University of Catania, Viale A. Doria 6, 95125 Catania, Italy
[4] Department of Agriculture, Food and Environment, University of Catania, Via Valdisavoia 5, 95123 Catania, Italy; donata.arena@unict.it
* Correspondence: francesco.attanasio@cnr.it (F.A.); barbara.tomasello@unict.it (B.T.)
† These authors contributed equally to this work.

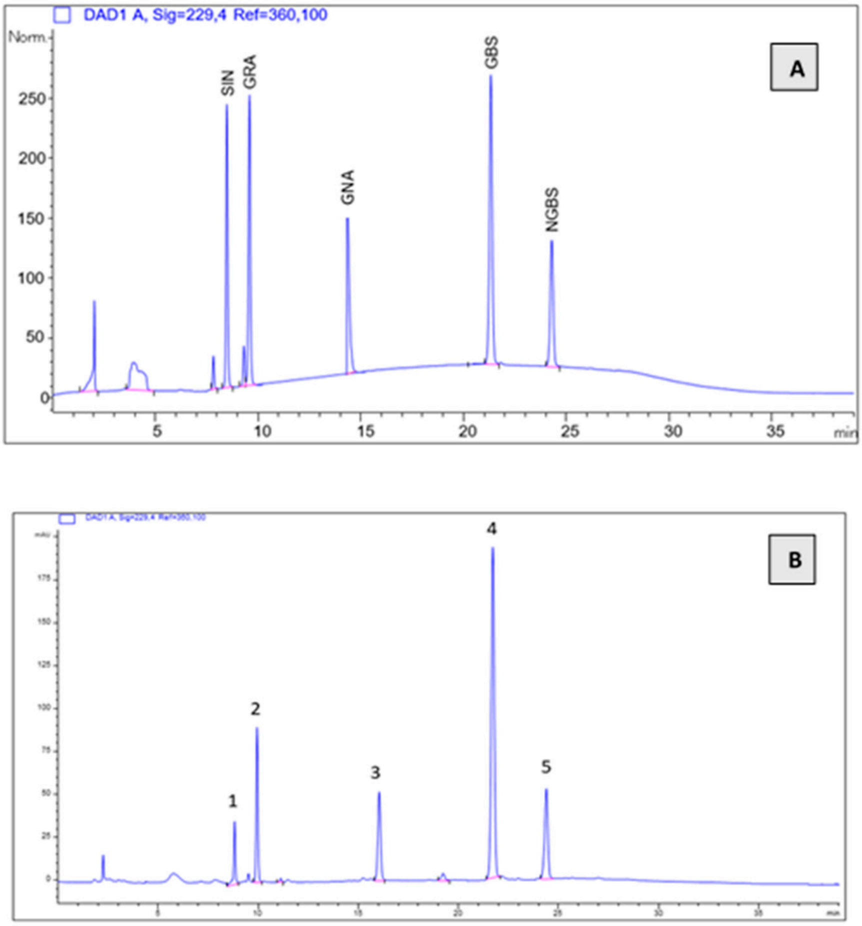

Figure S1. HPLC-DAD chromatograms of: A) desulfo-GLSs standards SIN (sinigrin), GRA (glucoraphanin), GNA (gluconapin), GBS (glucobrassicin), NGBS (neoglucobrassicin); B) desulfo-

GLSs in *Brassica oleracea* var. *italica* (BE): 1 (SIN, sinigrin), 2 (GRA, glucoraphanin), 3 (GNA, gluconapin), 4 (GBS, glucobrassicin), 5 (NGBS, neoglucobrassicin).

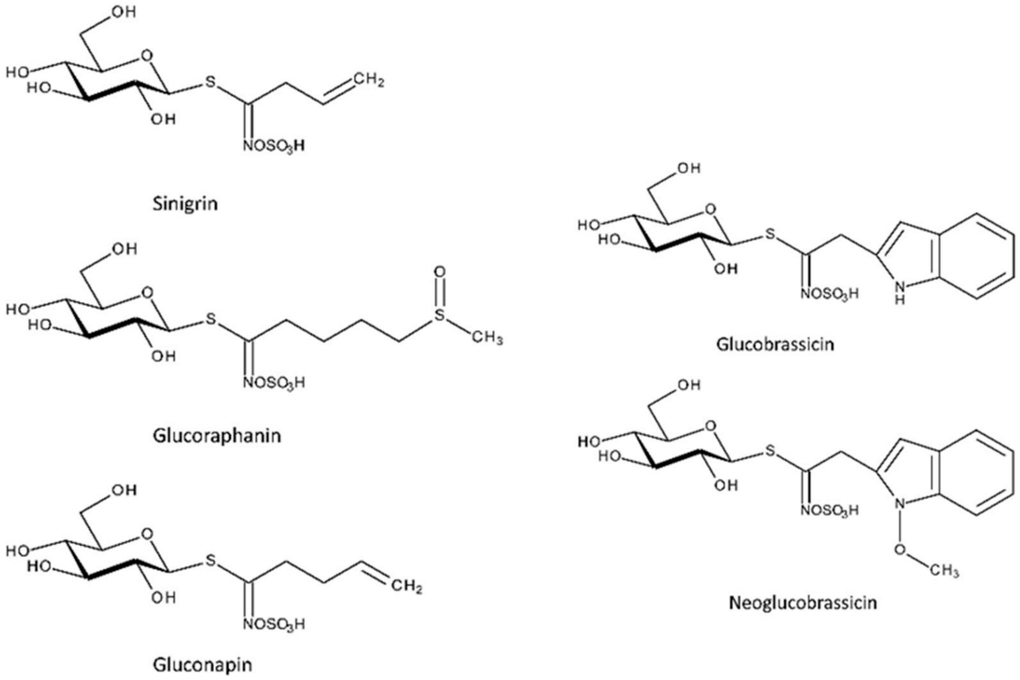

Figure S2: Chemical structures of identified compounds in BE designed with BIOVIA Draw (https://discover.3ds.com/biovia-draw-academic)

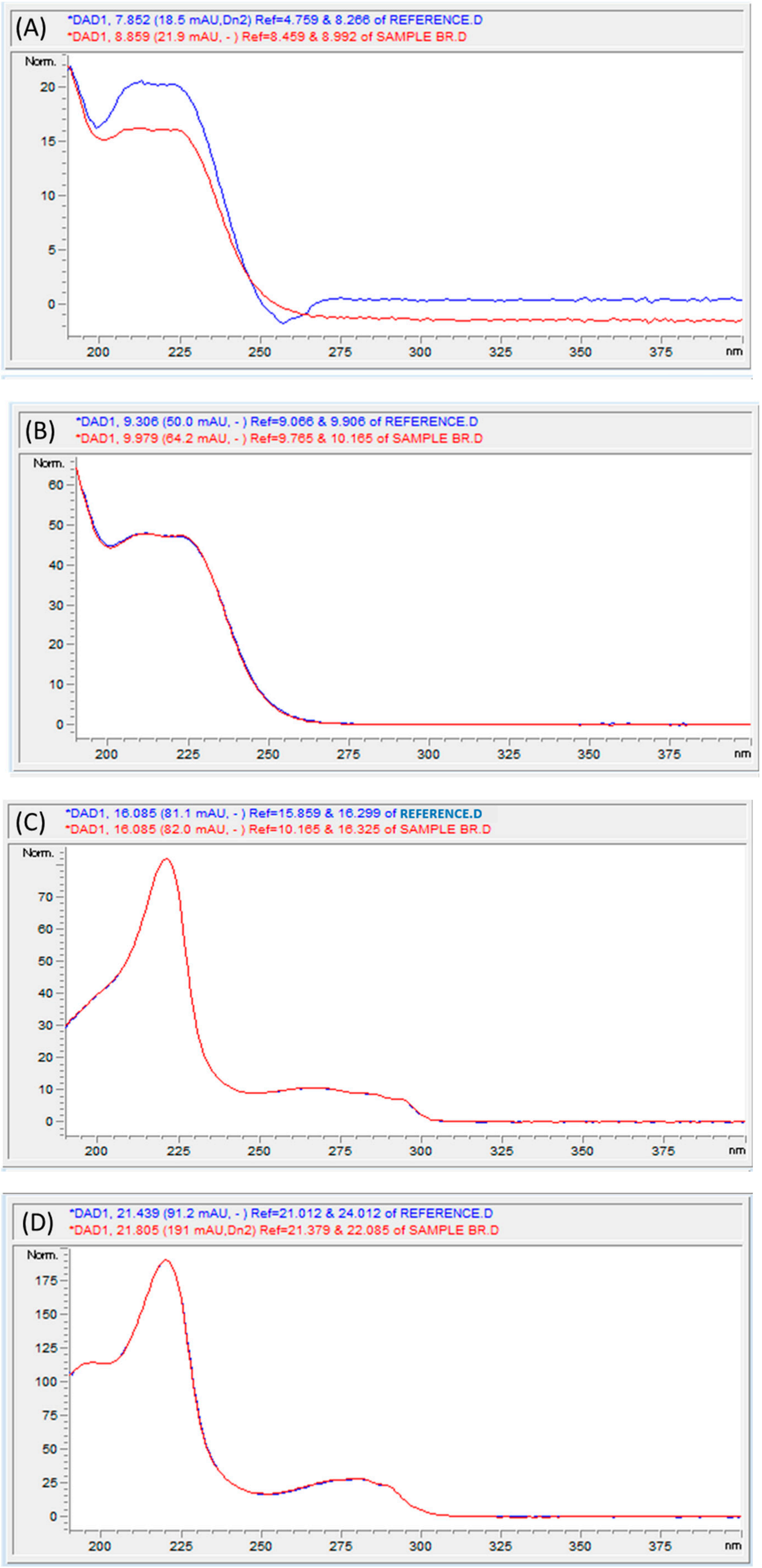
(A)
*DAD1, 7.852 (18.5 mAU,Dn2) Ref=4.759 & 8.266 of REFERENCE.D
*DAD1, 8.859 (21.9 mAU, -) Ref=8.459 & 8.992 of SAMPLE BR.D
Norm.
20
15
10
5
0
200
225
250
275
300
325
350
375
nm
(B)
*DAD1, 9.306 (50.0 mAU, -) Ref=9.066 & 9.906 of REFERENCE.D
*DAD1, 9.979 (64.2 mAU, -) Ref=9.765 & 10.165 of SAMPLE BR.D
Norm.
60
50
40
30
20
10
0
200
225
250
275
300
325
350
375
nm
(C)
*DAD1, 16.085 (81.1 mAU, -) Ref=15.859 & 16.299 of REFERENCE.D
*DAD1, 16.085 (82.0 mAU, -) Ref=10.165 & 16.325 of SAMPLE BR.D
Norm.
70
60
50
40
30
20
10
0
200
225
250
275
300
325
350
375
nm
(D)
*DAD1, 21.439 (91.2 mAU, -) Ref=21.012 & 24.012 of REFERENCE.D
*DAD1, 21.805 (191 mAU,Dn2) Ref=21.379 & 22.085 of SAMPLE BR.D
Norm.
175
150
125
100
75
50
25
0
200
225
250
275
300
325
350
375
nm

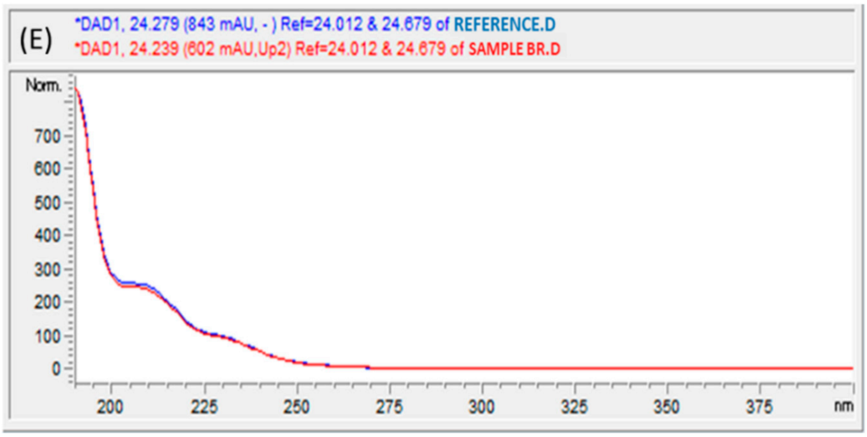

Figure S3: UV spectra of the standard compound alone (blue line) or identified in BE (red line) recorded at 230 nm: sinigrin (A), glucoraphanin (B), gluconapin (C), glucobrassicin (D), neoglucobrassicin (E)
